# A pilot study of brain morphometry following donepezil treatment in mild cognitive impairment: volume changes of cortical/subcortical regions and hippocampal subfields

**DOI:** 10.1038/s41598-020-67873-y

**Published:** 2020-07-02

**Authors:** Gwang-Won Kim, Byeong-Chae Kim, Kwang Sung Park, Gwang-Woo Jeong

**Affiliations:** 1 Advanced Institute of Aging Science, Chonnam National University, Gwangju, 61186 Republic of Korea; 2 Department of Psychiatry, Massachusetts General Hospital and Harvard Medical School, Boston, 02129 USA; 3 Department of Neurology, Chonnam National University Medical School, Gwangju, 61469 Republic of Korea; 4 Department of Urology, Chonnam National University Hospital, Chonnam National University Medical School, Gwangju, 61469 Republic of Korea; 5 Department of Radiology, Chonnam National University Hospital, Chonnam National University Medical School, 42 Jebong-Ro, Donggu, Gwangju, 61469 Republic of Korea

**Keywords:** Neuroscience, Anatomy

## Abstract

The efficacy of donepezil is well known for improving the cognitive performance in patients with mild cognitive impairment (MCI) and Alzheimer’s disease (AD). Most of the recent neuroimaging studies focusing on the brain morphometry have dealt with the targeted brain structures, and thus it remains unknown how donepezil treatment influences the volume change over the whole brain areas including the cortical and subcortical regions and hippocampal subfields in particular. This study aimed to evaluate overall gray matter (GM) volume changes after donepezil treatment in MCI, which is a prodromal phase of AD, using voxel-based morphometry. Patients with MCI underwent the magnetic resonance imaging (MRI) before and after 6-month donepezil treatment. The cognitive function for MCI was evaluated using the questionnaires of the Korean version of the mini-mental state examination (K-MMSE) and Alzheimer’s disease assessment scale-cognitive subscale (ADAS-Cog). Compared with healthy controls, patients with MCI showed significantly lower GM volumes in the hippocampus and its subfields, specifically in the right subiculum and left cornu ammonis (CA3). The average scores of K-MMSE in patients with MCI improved by 8% after donepezil treatment. Treated patients showed significantly higher GM volumes in the putamen, globus pailldus, and inferior frontal gyrus after donepezil treatment (p < 0.001). However, whole hippocampal volume in the patients decreased by 0.6% after 6-month treatment, and the rate of volume change in the left hippocampus was negatively correlated with the period of treatment. These findings will be useful for screening and tracking MCI, as well as understanding of the pathogenesis of MCI in connection with brain morphometric change.

## Introduction

Alzheimer’s disease (AD) is generally divided into 3 stages: preclinical AD, mild cognitive impairment (MCI) due to AD pathology, and AD-dementia. MCI is a prodromal stage of AD, which leads to expected cognitive decline of normal aging or other brain dysfunction^1,2^. AD is a chronic brain disorder associated with degeneration of cholinergic neurons and the progressive development of dementia and it is characterized by the presence of neurofibrillary tangles and senile plaques, impaired synaptic function, and cell los^2,3^. The key clinical symptom of AD is a progressive deterioration in learning and memory ability due to a deficiency of acetylcholine (ACh) levels in the brain^3^. Acetylcholine is an essential neurotransmitter in the brain, which can limit cognitive function when present in synapses at decreased levels. Acetylcholinesterase inhibitors (AChEIs) are employed to reduce the rate at which ACh is broken down, thereby increasing the concentration of ACh at the synapses and presynaptic receptors. The AChEIs offsets the loss of ACh caused by the death of cholinergic neurons^4^. Hence, treatment of patients with MCI with AChEIs prevents the breakdown of ACh and increases cholinergic transmission, leading to the amelioration of symptoms.

To date, four AChEIs (donepezil, galantamine, rivastigmine, and tacrine) have been approved by the United States Food and Drug Administration (FDA) for AD treatment, which are capable of ameliorating cognitive decline by inhibiting acetylcholinesterase activity^3^. Among these, three (donepezil, galantamine, and rivastigmine) have similar effects, but donepezil is the most frequently prescribed^5^. Donepezil inhibits acetylcholinesterase activity in the cerebral cortex, hippocampus, and striatum of the rat brain, producing increased ACh activity in the brai^6,7^. Several other studies^8,9^ have supported this evidence by showing that donepezil treatment is clinically efficient to improve cognitive performance in patients with AD.

In general, a progressive hippocampal volume loss is accompanied by a neuro-functional change which is an important characteristic of AD. Specifically, the hippocampal volume change is most useful for both diagnosing and tracking AD^10,11^. Therefore, the identification of morphological changes of the brain by the use of magnetic resonance imaging (MRI) is increasingly important in the study of neurological and psychiatric diseases^12–14^. Several MRI studie^3,11^ evaluated the effects of AD treatments with donepezil regimen in connection with morphological changes. Hashimoto et al.^3^ reported that donepezil treatment slows down the progression of hippocampal atrophy in patients with AD, suggesting a neuroprotective effect of donepezil in this disease. However, Wang et al.^15^ has suggested that donepezil treatment did not affect the hippocampal volume change in mild AD.

Until now, there have been no picture of how donepezil treatment influences the hippocampal subfields in patients with MCI. Thus, this study aimed to evaluate the gray matter volume changes over the cortical/subcortical regions, especially, focusing on the hippocampal subfields in the connection with donepezil treatment in MCI.

## Subjects and method

### Ethics

This study was approved by the Institutional Review Board of Chonnam National University Hospital (IRB-CNUH). Before MR scanning, the experimental procedure was explained to all volunteers and written informed consent was obtained. Also, all the experimental procedure and method were performed in accordance with the relevant guidelines and regulations approved by IRB-CNUH.

### Subjects

Ten patients with MCI (male:female = 4:6, mean age = 72.4 ± 7.9 years) and 9 age-matched healthy controls (male:female = 3:6, mean age = 70.7 ± 3.5 years) participated in this study. Ten patients with MCI were recruited by the following criteria: first, MCI of Alzheimer-type by the criteria of both the DSM-IV and the National Institute of Neurological and Communicative Diseases and Stroke-Alzheimer Disease and Related Disorders Association (NINCDS-ADRDA), second, a score less than 26 on the Korean version of the Mini-Mental State Examination (K-MMSE), third, a score of 0.5 or 1 on the Clinical Dementia Rating (CDR), fourth, no history of MCI treatment and other neurological or psychiatric illnesses, fifth, reconfirmation by the typical symptom severity including change in cognition recognized by the affected individual or observers, objective impairment in one or more cognitive domains, independence in functional activities, and absence of dementia^16^. Here, it should be noted that the heterogeneous nature of the cognitive impairment was not considered in the current study. Nine healthy controls were selected by the following criteria: first, no MCI by the criteria of both the DSM-IV and the NINCDS-ADRDA, second, no history of AChEI treatment and neurological or psychiatric disorders.

Ten patients underwent the MR examinations before (baseline) and after (follow-up) donepezil treatment (Table 1). After performing the 1st MR examination, the 10 patients received 5 mg/day of Aricept (donepezil hydrochloride; Pfizer Inc., New York, NY) for the first 28 days and 10 mg/day thereafter. Aricept was approved by the United States FDA for Alzheimer’s disease in 2004. The dose of Aricept used in this study was equal to that to treat patients with MCI in Chonnam National University Hospital. The treatment duration for the patients was 192.5 ± 29.6 days. During that period, all patients had no side effects like agitation, gastrointestinal bleeding, stomach ulcers, and so on.

**Table 1:** Demographic and clinical characteristics of patients with MCI (baseline), donepezil-treated patients (follow-up), and healthy controls (HC).

	MCI patients			Healthy control (HC, n = 9)	Statistical analysis (p-value)		
	Baseline (n = 10)	Follow-up (n = 10)	Mean rate after treatment		Baseline vs. Follow-up	HC vs. Baseline	HC vs. Follow-up
Age (years)	72.4 ± 7.9	72.4 ± 7.9	–	70.7 ± 3.5	–	p = 0.545	p = 0.545
K-MMSE	16.3 ± 4.9	17.6 ± 3.5	+ 8.0%	28.6 ± 1.1	p = 0.031^a^	p = 0.0002^b^	p = 0.0002^b^
ADAS-Cog	25.3 ± 8.0	25.1 ± 6.1	− 0.8%	–	p = 0.759	–	–
CDR	0.7 ± 0.2	0.6 ± 0.2	− 14.3%	–	p = 0.157	–	–
GDS	13.0 ± 5.0	12.6 ± 4.8	− 3.1%	–	p = 0.370	–	–

^a^Significant difference (Wilcoxon's signed-ranks; *p* < 0.05) between MCI patients (baseline) and treated patients (follow-up).

^b^Significant differences (Mann–Whitney *U*; *p* < 0.001) in both "healthy controls vs. MCI patients" and "healthy controls vs. treated patients".

K-MMSE, Korean version of the mini-mental state examination; ADAS-Cog, AD assessment scale-cognitive subscale; CDR, clinical dementia rating; GDS, geriatric depression scale.

The MCI symptom severity was evaluated in the groups receiving and not receiving donepezil treatment using the questionnaires of the K-MMSE, the AD assessment scale-cognitive subscale (ADAS-Cog), the CDR scale, and the geriatric depression scale (GDS). A Wilcoxon's signed-ranks test was used to compare scores on the K-MMSE, ADAS-Cog, CDR, and GDS between patients receiving and not receiving donepezil treatment. The contrasts of “healthy controls vs. untreated patients with MCI”, “healthy controls vs. donepezil-treated patients with MCI” and “untreated patients with MCI vs. donepezil-treated patients with MCI” were analyzed with a Mann–Whitney *U*-test.

### MR imaging

The MRI study was performed on a 3.0-T Magnetom Tim Trio MR Scanner (Siemens Medical Solutions, Erlangen, Germany) with an 8-channel receiver head coil of birdcage type. Ten untreated patients with MCI and ten treated patients with MCI underwent MRI and MRS. The T1-weighted sagittal images were acquired using a three dimensional magnetization-prepared rapid-acquisition gradient echo (3D-MPRAGE) pulse sequence with a repetition time (TR)/echo time (TE) = 1,700 ms/2.2 ms, field of view (FOV) = 256 × 256 mm^2^, and matrix = 512 × 512.

### Data processing and analysis

MRI data was analyzed using SPM8 software (Statistical Parametric Mapping, Wellcome Department of Cognitive Neurology, London, U.K.) with diffeomorphic anatomical registration through exponentiated Lie algebra (DARTEL) analysis. Prior to data processing, all individual data were aligned to the anterior-to-posterior commissure line on the transverse plane. After correction of bias in the images due to field non-uniformity, MRI data was segmented into gray matter (GM), white matter (WM), and cerebrospinal fluid (CSF) using tissue probability maps based on the International Consortium of Brain Mapping (ICBM) space template for the East Asian brain typ^17^. The mean templates for GM and WM were created using individual GM and WM images. All the images were normalized to the Montreal Neurological Institute template and were subsequently separated into GM and WM images. The images were then smoothed with a 8-mm full-width-at-half-maximum (FWHM) isotropic Gaussian kernel. For the group analysis, a paired t-test was used to compare GM volumes before and after donepezil treatment in patients with MCI. A two-sample t-test, with age, sex, and intracranial volume (ICV) as covariates, was used to compare GM volumes between healthy controls and patients in the Statistical nonParametric Mapping (SnPM13). The result was thresholded at a cluster level corrected threshold of p < 0.05 (n = 5,000 permutations, family-wise error (FWE)-corrected) with a cluster-determining threshold at the voxel level p < 0.001. Based on findings from the previous studies focusing on MCI and AD^10,18–20^, brain regions of interest (ROIs) were selected as follows: frontal, parietal, temporal, and occipital cortices, and subcortical regions. GM volume and Montreal Neurological Institute coordinates were analyzed using the SPM8 and MRIcron software^13,21^.

**Figure 1:**
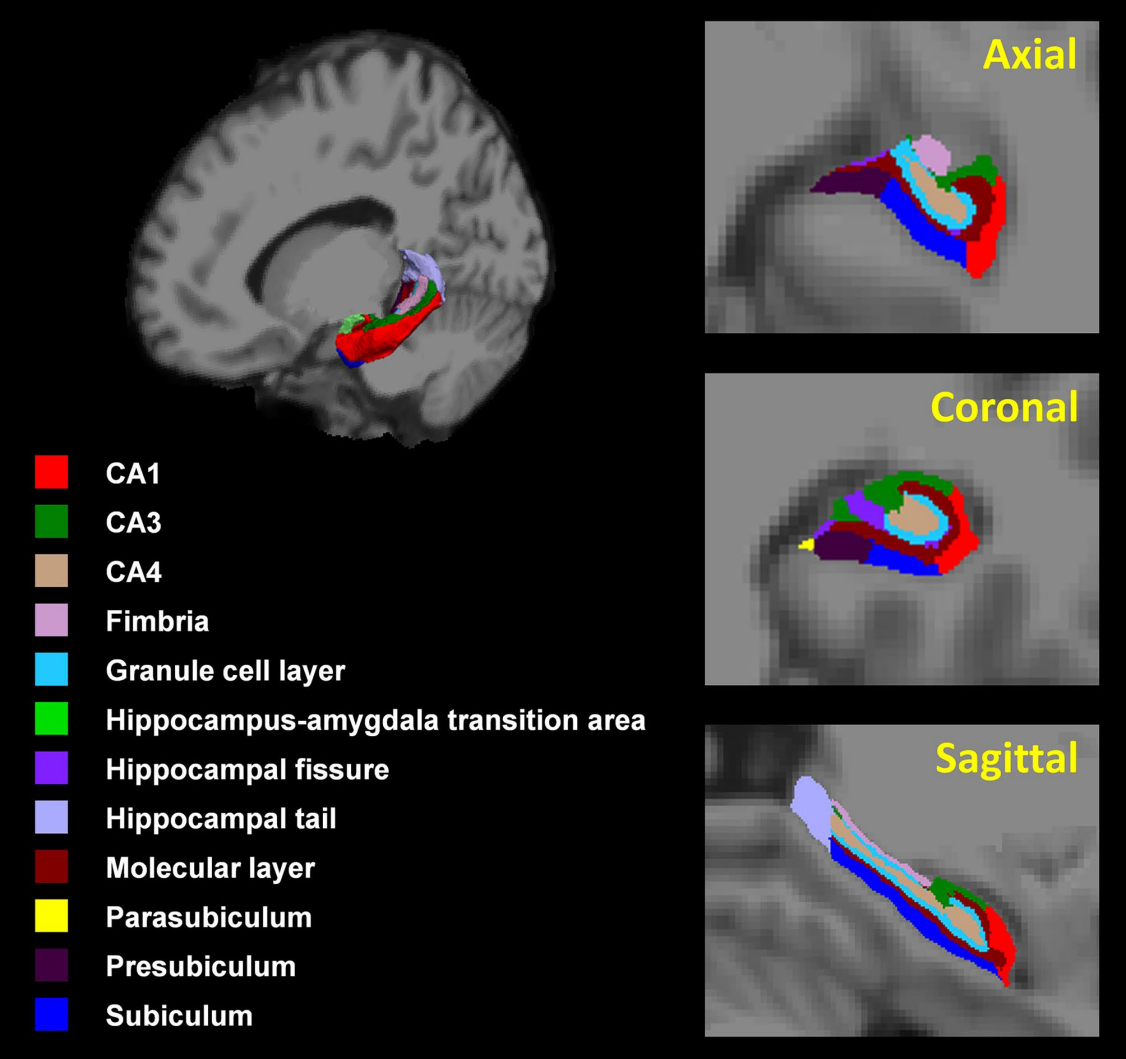
3D atlas of the hippocampus and hippocampal subfield segmentation. CA, cornu ammonis. This figure was created using Freesurfer (version 6.0 https://surfer.nmr.mgh.harvard.edu) and Microsoft Powerpoint (version 16 https://www.microsoft.com).

Hippocampal subfields were calculated using the FreeSurfer v6 software (MGH, Boston, MA, USA). This procedure is described in detail in previous papers^20,22^. Post-processing of images comprised the following steps: correction for head motion and non-uniformity of intensity, Talairach transformation of each subject’s brain, removal of non-brain tissue, segmentation of cortical gray, subcortical white and deep GM volumetric structures, triangular tessellation of the GM/WM matter interface and GM/CSF boundary, and topology correction. Then, automated segmentation of the hippocampal subfields was performed using a built-in module of FreeSurfer^20,22^. Twelve hippocampal subfields were extracted for each hemisphere, including the hippocampal tail, subiculum, cornu ammonis (CA)1, hippocampal fissure, presubiculum, parasubiculum, molecular layer of the hippocampus, granular cells layer of the dentate gyrus (GC-DG), CA3, CA4, fimbria, and hippocampus-amygdala transition area (HATA) (Fig. 1). The multivariate analysis of variance, with age, sex, and ICV as covariates, was used to compare the hippocampal subfield volumes between healthy controls and untreated or treated patients, and the paired t-test was used to compare the hippocampal subfield volumes before and after donepezil treatment in patients with MCI. A Bonferroni correction for FWE at alpha level (p < 0.05) was applied. The rate of volume change between baseline and follow-up in MCI patients was calculated by the equation, (Volume_follow-up_ − Volume_baseline_)/(Volume_baseline_). Correlations between the rate of volume change in the hippocampus and the period of treatment in patients were analyzed using a Spearman’s correlation test. Statistical analysis was performed using SPSS (version 25.0, IBM, Armonk, NY, USA).

## Results

### Changes in symptom severity

The average K-MMSE scores in healthy controls (n = 9), untreated patients (n = 10, baseline), and donepezil-treated patients (n = 10, follow-up) were 28.6 ± 1.1, 16.3 ± 4.9, and 17.6 ± 3.5, respectively (Table 1). We found a significant difference in the K-MMSE (p = 0.031) between untreated patients and donepezil-treated patients, in which the average scores were improved following donepezil treatment. Also, it should be noted that the average scores on the ADAS-Cog in untreated and donepezil-treated patients were 25.3 ± 8.0 and 25.1 ± 6.1, respectively (p = 0.759, NS); the average scores on the CDR in both groups were 0.7 ± 0.2 and 0.6 ± 0.2, respectively (p = 0.157, NS) and the scores on the GDS were 13.0 ± 5.0 and 12.6 ± 4.8, respectively (p = 0.370, NS) (Table 1).

### Global GM volume changes in three groups

**Figure 2:**
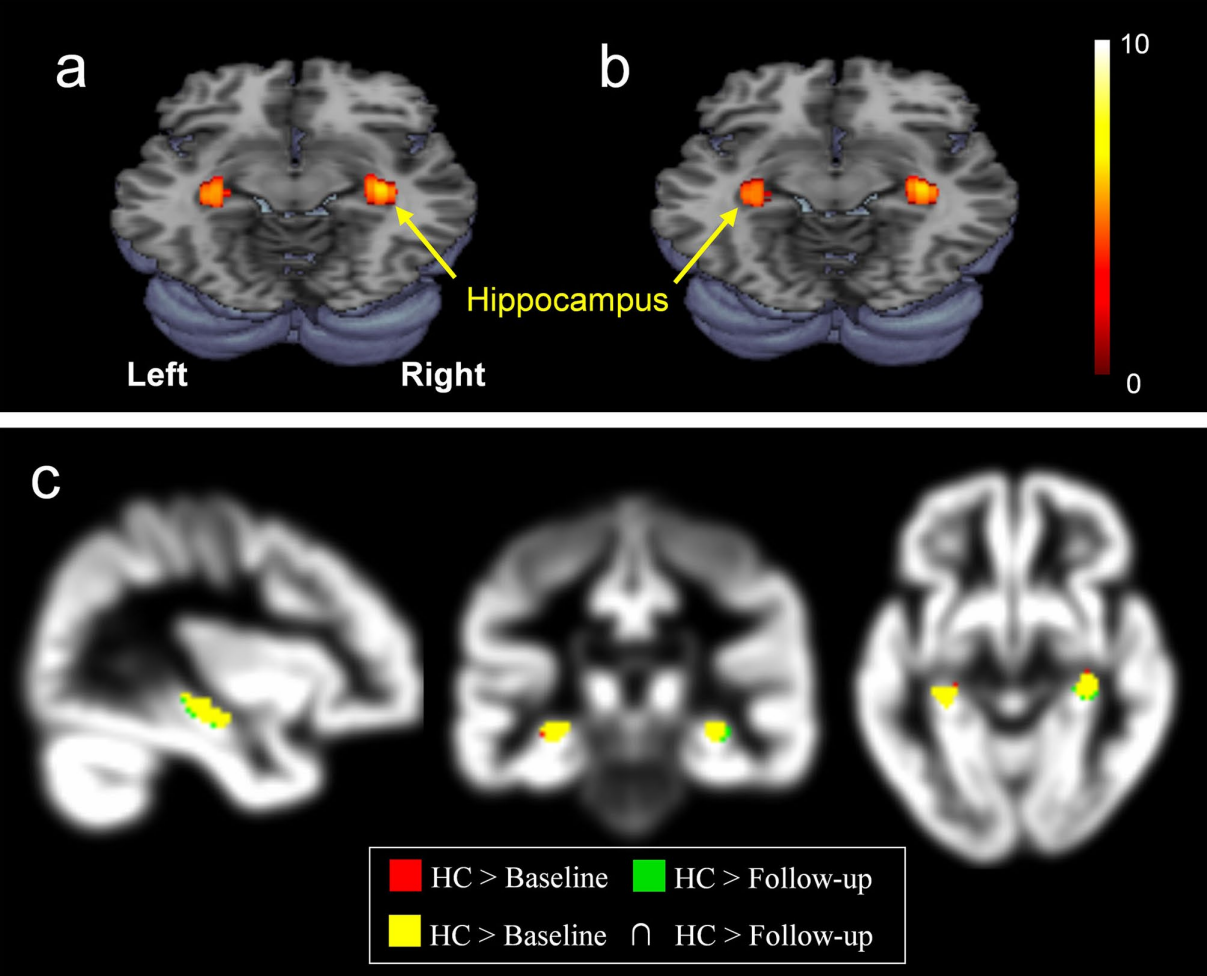
Demonstration of the decreased gray matter volumes of the hippocampus: (**a**) untreated patients (baseline) relative to healthy control (HC), (**b**) treated patients (follow-up) relative to healthy controls (FWE corrected, p < 0.05), and (**c**) intersection set of {HC > baseline} and {HC > follow-up} which highlights the overlapping areas with different colors on the sagittal, coronal, and axial planes, i.e. green, HC > baseline; red, HC > follow-up; yellow, the intersection of the two sets, red and green. This figure was created using SPM (version 8 https://www.fil.ion.ucl.ac.uk/spm), MRIcron (version 6 https://www.nitrc.org/projects/mricron), and Microsoft Powerpoint (version 16 https://www.microsoft.com).

**Figure 3:**
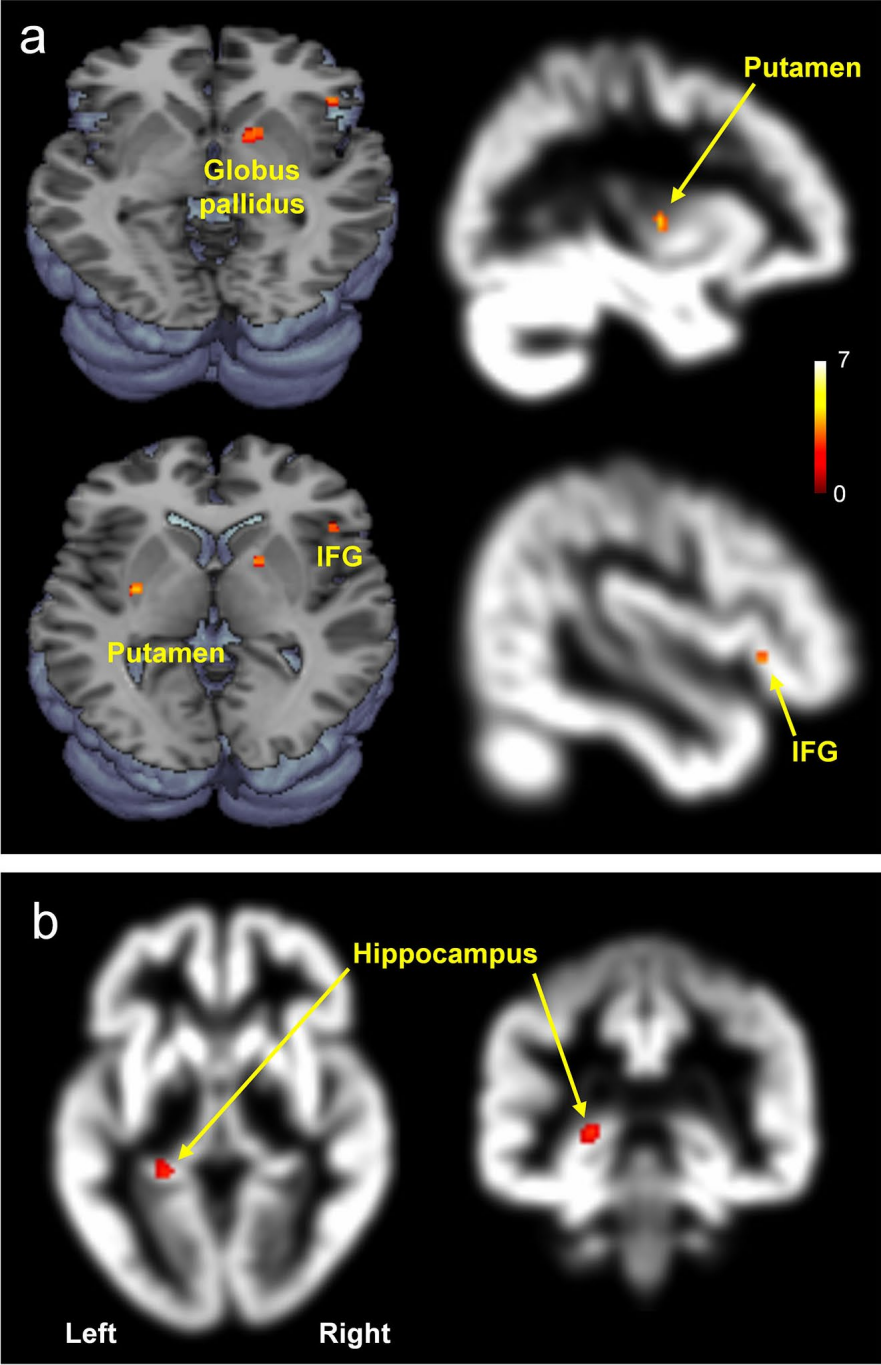
Brain regions with significantly increased GM volumes (a) and decreased GM volumes (**b**) in donepezil-treated patients compared with untreated patients [p < 0.001 (**a**) or p < 0.05 (**b**)]. IFG, inferior frontal gyrus. This figure was created using SPM (version 8 https://www.fil.ion.ucl.ac.uk/spm), MRIcron (version 6 https://www.nitrc.org/projects/mricron), and Microsoft Powerpoint (version 16 https://www.microsoft.com).

Compared with healthy controls (n = 9), both of the untreated (n = 10) and treated (n = 10) patients showed significantly reduced GM volumes in the bilateral hippocampus (FWE corrected, p < 0.05, Fig. 2). As for the comparison between untreated and treated patients with 6-month donepezil therapy, the treated patients showed significantly higher GM volumes in the putamen, globus pailldus, and inferior frontal gyrus after (uncorrected; p < 0.001; Fig. 3a), and concurrently showed lower GM volume in the left hippocampus (uncorrected; p < 0.05; Fig. 3b).

### Hippocampal and its subfield volume changes

Volumes of the hippocampus in healthy controls, untreated patients, and donepezil-treated patients were 7,724.8 ± 513.6, 6,312.1 ± 731.4, and 6,277.0 ± 654.5 mm^2^, respectively (HC vs. baseline: p = 0.006, HC vs. follow-up: p = 0.004, baseline vs. follow-up: p = 0.721). The average hippocampal volumes in the untreated and treated patients are lower than that in healthy controls. However, the hippocampal volumes of untreated patients and treated patients were not significantly different from each other.

**Figure 4:**
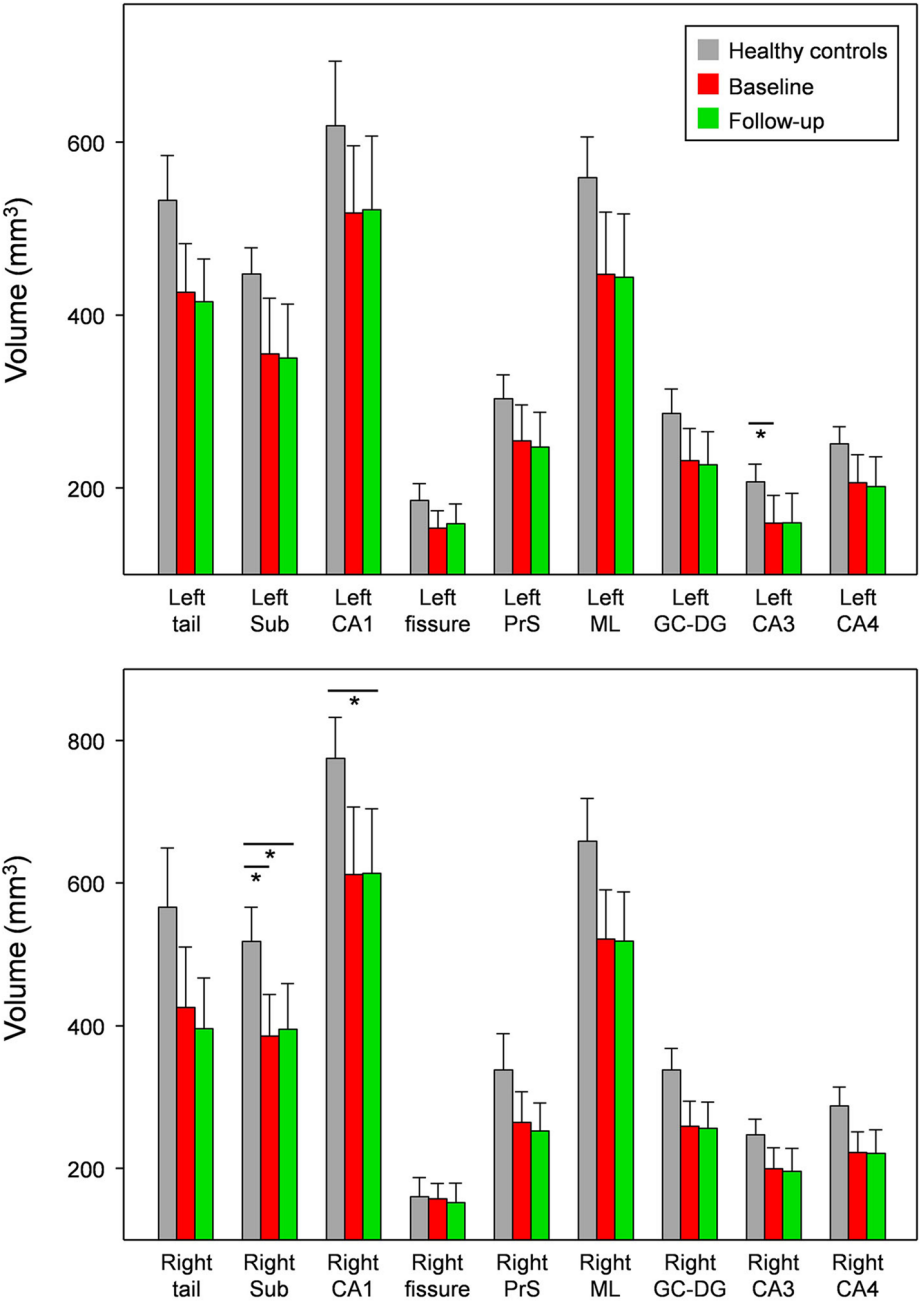
Comparison of the volumes of the hippocampal subfields in healthy controls, untreated patients (baseline), and treated patients (follow-up). Sub, subiculum; CA, cornu ammonis, PrS, presubiculum; ML, molecular layer of the hippocampus, GC-DG, granular cells layer of the dentate gyrus. This figure was created using SigmaPlot (version 13 https://systatsoftware.com/products/sigmaplot) and Microsoft Powerpoint (version 16 https://www.microsoft.com). *Significant difference (Bonferroni corrected, p < 0.05).

**Figure 5:**
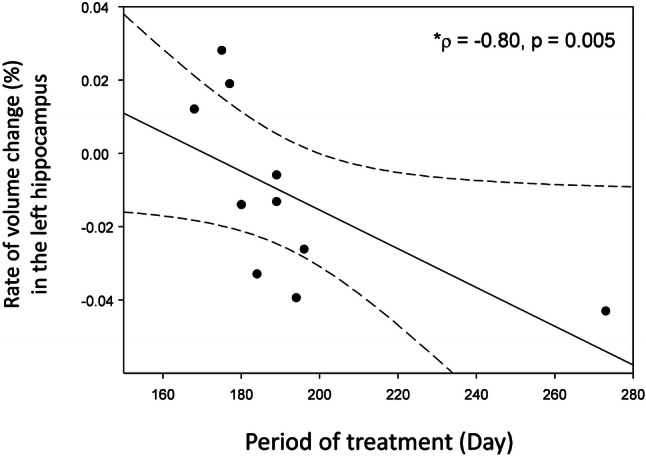
The rate of volume change in the left hippocampus is negatively correlated with the period of treatment in patients with MCI (Spearman’s rho = − 0.80, p = 0.005). Dotted lines show 95% CI. The rate of volume change between baseline and follow-up in MCI patients was calculated by the equation, (Volume_follow-up_ − Volume_baseline_)/(Volume_baseline_). This figure was created using SigmaPlot (version 13 https://systatsoftware.com/products/sigmaplot) and Microsoft Powerpoint (version 16 https://www.microsoft.com).

As shown in Fig. 4, the untreated patients showed significantly reduced volumes in the right subiculum and left CA3 compared with healthy controls (Bonferroni corrected, p < 0.05, Table 2). The treated patients also showed reduced volumes in the right subiculum and right CA1 compared with healthy controls (Bonferroni corrected, p < 0.05). However, the hippocampal subfield volumes of untreated patients and treated patients were not significantly different (Table 3). Interestingly, the rate of volume change in the left hippocampus in untreated patients was negatively correlated with the period of treatment (rho = -0.80, p = 0.005, Fig. 5).

**Table 2:** Comparison of hippocampal subfield volumes between healthy controls (HC) and patients with MCI (baseline).

Subfields	Healthy controls (HC)	MCI patients (baseline)	Volume difference (baseline—HC)	F-value	P-value	Cohen's d
L tail	533.9 ± 52.0	427.5 ± 56.5	− 106.3	8.6	0.010	1.42
R tail	567.4 ± 83.2	427.0 ± 84.8	− 140.4	5.9	0.027	1.18
L subiculum	448.5 ± 30.6	356.1 ± 64.4	− 92.4	5.4	0.034	1.13
R subiculum	519.7 ± 47.8	387.0 ± 58.3	− 132.7	14.8	0.001*	1.87
L CA1	620.1 ± 74.5	519.1 ± 78.0	− 101.0	3.7	0.071	0.93
R CA1	776.1 ± 57.8	613.5 ± 94.7	− 162.6	11.9	0.003	1.68
L fissure	186.7 ± 19.6	155.0 ± 19.8	− 31.6	5.9	0.027	1.18
R fissure	161.8 ± 26.4	158.7 ± 21.8	− 3.1	0.6	0.446	0.38
L presubiculum	304.5 ± 27.5	255.8 ± 41.6	− 48.8	3.7	0.074	0.93
R presubiculum	338.8 ± 51.6	266.0 ± 42.6	− 72.8	6.2	0.025	1.21
L molecular layer	560.2 ± 47.3	448.5 ± 71.9	− 111.8	7.5	0.015	1.33
R molecular layer	659.9 ± 60.3	522.9 ± 69.0	− 137.0	10.3	0.006	1.56
L GC-DG	287.4 ± 28.5	232.6 ± 37.2	− 54.8	6.9	0.018	1.28
R GC-DG	339.3 ± 30.4	260.1 ± 35.6	− 79.1	7.8	0.013	1.36
L CA3	208.1 ± 20.6	160.7 ± 31.8	− 47.4	12.5	0.003*	1.72
R CA3	248.4 ± 21.8	200.8 ± 29.4	− 47.5	6.3	0.023	1.22
L CA4	252.0 ± 20.1	207.0 ± 32.8	− 45.0	9.6	0.007	1.51
R CA4	288.8 ± 26.7	223.5 ± 29.0	− 65.3	7.7	0.014	1.35

Mean and standard deviation of hippocampal subfield volumes in mm^3^.

*CA* cornu ammonis, *GC-DG* granular cells layer of the dentate gyrus.

*Bonferroni correction suggests an appropriate level of p < 0.003.

**Table 3:** Comparison of hippocampal subfield volumes between patients with MCI (baseline) and treated patients (follow-up).

Subfields	MCI patients (baseline)	Treated patients (follow-up)	Volume difference (Follow-up—baseline)	Rate of volume change (follow-up—baseline)^a^ (%)	p-value
L tail	427.5 ± 56.5	416.6 ± 49.5	− 10.9	− 2.5	0.017
R tail	427.0 ± 84.8	397.4 ± 71.0	− 29.6	− 6.9	0.386
L subiculum	356.1 ± 64.4	351.6 ± 62.7	− 4.5	− 1.2	0.139
R subiculum	387.0 ± 58.3	396.5 ± 64.2	9.5	2.4	0.646
L CA1	519.1 ± 78.0	522.8 ± 85.1	3.7	0.7	0.333
R CA1	613.5 ± 94.7	615.0 ± 90.2	1.5	0.2	0.959
L fissure	155.0 ± 19.8	159.8 ± 22.7	4.8	3.0	0.203
R fissure	158.7 ± 21.8	153.3 ± 27.4	− 5.4	− 3.3	0.508
L presubiculum	255.8 ± 41.6	248.4 ± 40.2	− 7.4	− 2.8	0.169
R presubiculum	266.0 ± 42.6	253.9 ± 39.2	− 12.1	− 4.5	0.203
L molecular layer	448.5 ± 71.9	445.0 ± 73.4	− 3.5	− 0.7	0.445
R molecular layer	522.9 ± 69.0	520.0 ± 68.8	− 2.9	− 0.5	0.959
L GC-DG	232.6 ± 37.2	228.1 ± 38.3	− 4.5	− 1.9	0.333
R GC-DG	260.1 ± 35.6	257.2 ± 37.2	− 2.9	− 1.1	0.721
L CA3	160.7 ± 31.8	161.0 ± 33.9	0.3	0.2	0.959
R CA3	200.8 ± 29.4	197.2 ± 32.3	− 3.6	− 1.8	0.878
L CA4	207.0 ± 32.8	202.9 ± 34.4	− 4.1	− 1.9	0.241
R CA4	223.5 ± 29.0	222.6 ± 32.7	− 0.9	− 0.3	0.878

Mean and standard deviation of hippocampal subfield volumes in mm^3^.

*CA* cornu ammonis, *GC-DG* granular cells layer of the dentate gyrus.

*(Follow-up − Baseline)/Baseline × 100.

## Discussion

MRI-based volumetry of the hippocampus has been proposed as a useful tool for the clinical diagnosis of AD^3,23^. Among the characteristic neuropathological changes seen in AD, the most prominent structural changes at the initial stage occur in hippocampal formation^3,24^. In our study, both of the patients with MCI showed GM atrophy in the lateral hippocampus compared with healthy controls. We also found that the untreated patients had significantly lower scores on the K-MMSE as compared with healthy controls.

Figure 4 compared the hippocampal subfield volumes between three groups. In comparison with healthy controls, the untreated patients showed significantly reduced volumes in the right subiculum and left CA3, whereas the treated patients showed reduced volumes in the right subiculum and right CA1. Although the treated patients showed reduced volumes in the left CA3 (p = 0.005), the p-value is not significant at the 0.5 level (p = 0.003) of Bonferroni correction. The hippocampal subfields atrophy has been shown to be implicated in the progression of AD. Especially, volume of the left subiculum was correlated with the performance score across auditory verbal learning test measures^20^. Also, atrophy in the subiculum and CA1 was strongly associated with AD diagnosis, and furthermore, such a strong correlation with hippocampal subfields is most predictive of future conversion from normal controls to MCI and MCI to AD^25^. Here, it is suggested that specific hippocampal subfield atrophy is closely related with MCI and/or early stages of AD.

Compared with untreated patients, the donepezil-treated patients showed significantly higher scores on the K-MMSE as well as increased GM volumes in the putamen, globus pallidus, and inferior frontal gyrus (Fig. 3). However, a volumetric increase attributable to donepezil treatment was not observed in the hippocampus, which is contrary to the finding in a previous study^11^. Nevertheless, Hashimoto et al.^3^ have suggested that the mean annual rate of hippocampal volume loss in donepezil-treated patients is significantly less than that in control patients. In our study, however, we did not find increased hippocampal subfield volumes in patients after 6 months of donepezil treatment. Also, the whole volume of the hippocampus volume decreased by 0.6% after 6 months of donepezil treatment. Also, note that the rate of volume change in the left hippocampus was negatively correlated with the period of treatment (Fig. 5).

Increases in the local GM volumes as seen in our study could be associated with a restoration of cell number induced by to donepezil treatment, an indirect neurotrophic effect on the neuropil, or both. Kotani et al.^26^ have suggested that donepezil activates central cholinergic transmission and thereby enhances the survival of newborn neurons in the dentate gyrus in a rat model. In another similar study^27^ with a rat model of vascular dementia demonstrated that the efficacy of donepezil treatment induced an increase of the expression of brain-derived neurotrophic factor (BDNF). Consistent with this finding, patients with early AD showed an increase in BDNF serum concentration after donepezil treatment^28^. The BDNF has been demonstrated to contribute to increased beta-amyloid degradation by promoting the expression of somatostatin^28,29^. Accumulations of beta-amyloid is one of the earliest changes in AD pathology and may cause neuronal death in the central nervous system (CNS)^3,30^. Preventing this is a second way in which BDNF may increase neuron numbers relative to untreated control patients. Moreover, BDNF belongs to the nerve growth factor family and plays important roles in neuronal survival, differentiation, and synaptic plasticity in the CNS^28^, suggesting that it may also expand neuropils by a neurotrophic effect.

In this current study, we found that compared with untreated patients, donepezil-treated group showed significantly increased GM volumes in the putamen, globus pallidus, and inferior frontal gyrus which are involved in improved cognitive function. A previous study^31^ demonstrated that, compared with a patient group with AD having a 2-point *increase* in the MMSE following nine month donepezil treatment, another patient group having a 2-point *decrease* showed significantly reduced GM volume in the putamen. In our study, however, donepezil-treated patients having a 1.3-point *increase* in the K-MMSE following six months treatment showed markedly increased GM volume of the same brain area in comparison to the untreated patients. The putamen is best known for its role in the planning and modulation of movement pathways, and is potentially involved in a variety of cognitive processes involving executive function, such as working memory^32^. In addition, putamen function is linked to a variety of neurotransmitters such as acetylcholine^33^, and this brain area is potentially associated with the improved cognitive function. Another important brain area showing sparing by donepezil treatment is the inferior frontal gyrus. De Lange et al.^34^ reported that patients with chronic fatigue syndrome showed significant increases of GM volume in the inferior frontal gyrus after cognitive behavioral treatment. Here, it is important to note that the relationship between specific GM volume increment and enhanced K-MMSE score in treated MCI patients might be not clearly explained because of the retest effect in K-MMSE.

This study has some limitations. First, the population of subjects is small, leading to a lower statistical power. To compensate this disadvantage, we used a statistical threshold of P value less than 0.05 using the Bonferroni correction, giving a statistically reliable significance level. Future studies with larger numbers of subjects are needed. Second, healthy control group was not followed up for the K-MMSE, ADAS-cog, CDR, and GDS after 6-month time frame equivalent to the treatment period. Third, we need further study on the comparison of the volume changes among mild vs. moderate vs. severe levels in AD with respect to the effects of donepezil treatment. Fourth, additional to brain morphometry, the combined multimodal imaging techniques including functional MR imaging, MR spectroscopy and positron emission tomography are needed to gain more valuable information on the functional activation and connectivity and cerebral metabolic changes in connection with therapeutic effect.

## Conclusion

This study has demonstrated variations of the gray matter volumes in the cortical and subcortical regions and hippocampal subfields after donepezil treatment in patients with MCI. Untreated MCI showed lower GM volumes in the hippocampus and its subfields, specifically in the right subiculum and left CA3 when comparing to healthy control. However, the hippocampal subfield volumes of untreated and treated patients were not significantly different from each other. These findings will be helpful to understand the pathogenesis of MCI as a prodromal phase of AD in connection with the morphometric changes of specific brain areas.

## Data Availability

The data that support the findings of this study are available from the corresponding author, Gwang-Woo Jeong, upon reasonable request.
